# Pattern, frequency and factors associated with inappropriate high dosing in chronic kidney disease patients at a tertiary care hospital in Pakistan

**DOI:** 10.1186/s12882-023-03167-5

**Published:** 2023-05-01

**Authors:** Muhammad Hayat, Nafees Ahmad, Syed Liaquat Ali Khan, Syed Mohkumuddin, Wajeeha Siddique, Amjad Khan, Muhammad Atif

**Affiliations:** 1 Department of Pharmacy Practice, Faculty of Pharmacy and Health Sciences, University of Balochistan, Quetta, Pakistan; 2 Department of Nephrology, Bolan Medical College, Quetta, Pakistan; 3 Department of Pharmacy, Quaid-i-Azam University, Islamabad, Pakistan; 4 Department of Pharmacy Practice, Faculty of Pharmacy, The Islamia University of Bahawalpur, Bahawalpur, Pakistan

**Keywords:** Age, antibiotics, antidiabetics, antihypertensives, Cardiovascular diseases, Chronic renal disease, Diabetes mellitus

## Abstract

**Background:**

Patients with chronic kidney diseases (CKD) are susceptible to the toxic drug effects if given unadjusted doses. Although Pakistan harbors a high burden of CKD patients, there is limited information available on the frequency, pattern and factors associated with unadjusted drug doses among CKD patients.

**Methods:**

This cross-sectional study conducted at Sandeman Provincial Hospital, Quetta included 303 non-dialysis ambulatory CKD patients (glomerular filtration rate < 60 ml/min/1.73m^2^). The patients’ data were collected through a purpose designed data collection form. The appropriateness of doses was checked against the renal drug handbook-2018, Kidney Disease Improving Global Outcome**s** guidelines, British National Formulary-2022, and manufacturer leaflets. Data were analysed by SPSS 23 and multiple binary logistic regression analysis was used to assess the factors associated with receiving inappropriate high doses. A p-value < 0.05 was considered statistically significant.

**Results:**

The patients received a total of 2265 prescription lines, with a median of eight different drugs per patient (interquartile range: 6–9 drugs). A total of 34.5% (783/2265) drugs required dose adjustment. Of these, doses were not adjusted for 56.1% (440) drugs in 162 (53.4%) patients. The most common pharmacological class of drugs requiring dose adjustment were antibiotics (79.1%), followed by antidiabetics (59.2%), diuretics (57.0%), angiotensin converting enzyme inhibitors (56.9%), beta blockers (56.9%), analgesics (56.0%), angiotensin receptor blockers (55.2%), domperidone (53.9%) and antihyperlipidmics (46.1%). Patient’s age of 41–60 (OR = 5.76) and > 60 years (OR = 9.49), hypertension (OR = 2.68), diabetes mellitus (OR = 3.47) and cardiovascular diseases (OR = 2.82) had statistically significant association (p-value < 0.05) with inappropriate high doses.

**Conclusion:**

The high frequency of inappropriate high doses suggests an important quality gap in medication dosing for patients with ND-CKD at the study site. Special attention should be paid to the drugs and patients with identified risk factors for receiving inappropriate high doses.

## Background

Chronic kidney disease (CKD) is characterized by a persistently low estimated glomerular filtration rate (eGFR) of less than 60 ml/min/1.73m^2^, typically present for more than three months [1, 2]. Due to reduced elimination of drugs and their metabolites in patients with CKD, there is an increased risk of potential toxicity and increased healthcare costs [3–5]. Patients with CKD often have multiple comorbidities and are prescribed various medications requiring dose adjustment, therefore, it is recommended to adjust the drug dosage based on the patient’s renal function [6]. Despite this recommendation, the research indicates that clinicians find it challenging to perform this dose adjustment. According to various published studies, the frequency of CKD patients receiving the inappropriate high doses reportedly ranges from 13 to 84.3% [7–11]. Independent variables commonly found to be associated with the reception of inappropriate high doses by CKD patients include CKD stage 4 & 5, polypharmacy, comorbidities and patients over 65 years of age [10–12]. By evaluating the dosage regimen and factors associated with inappropriate high doses among CKD patients, insights can be gained into the extent of the problem and the patients groups who are at high risk. The data from such studies can be used to design targeted interventions aimed at improving clinical practices, patient outcomes, and reduce healthcare costs associated with inappropriate dosing [13, 14].

Pakistan is the world’s sixth most populated country and harbors a high burden of CKD patients. A press talk by a nephrologist at Sheikh Zayed Hospital Lahore [15] and findings of a systematic review [16], suggest that over 20% of the country’s population suffers from CKD [17]. Despite this high prevalence of CKD patients, very little information is currently available from Pakistan about the frequency, the related factors, and drugs involved in inappropriate high dosing among CKD patients [10, 18]. Consequently, the purpose of this study was to address these gaps.

## Methods

### Study settings and design

The present study is a cross-sectional analytical study conducted at the nephrology outpatient department (OPD) of Sandeman Provincial Hospital (SPH) in Quetta, Balochistan. SPH is a large tertiary care hospital in the province, consisting of 800 beds and serving approximately 8000–10,000 patients daily [19, 20].

### Study patients

This study included established non-dialysis CKD (ND-CKD) patients who were at least 18 years old, suffered from CKD stage 3 and above, visited the nephrology OPD of SPH between November 1, 2020 and January 31, 2021 and were willing to participate in the study by giving written or oral consent (in case of uneducated patients). Patients with eGFR ≥ 60 ml/min/1.73m^2^, those undergoing haemodialysis, pregnant women and who had a history of renal transplant were excluded from the study. In order to avoid missing any patient with renal impairment, we used eGFR rather than serum creatinine cut off points for defining renal impairment. The eGFR of patients was calculated by using CKD-epi Eqs. [21, 22] and the eligible patients were categorized into CKD stage 3a (eGFR = 45–59 ml/min/1.73 m^2^), stage 3b (eGFR = 30–44 ml/min/1.73 m^2^), stage 4 (15–29 ml/min/1.73 m^2^) and stage 5 (< 15 ml/min/1.73 m^2^) [23].

To determine the minimum sample size for this study, we employed the Daniel’s sample size calculation formula (*n = Z*^*2*^*P (1 − P)/d*^*2*^) [25], where n = required sample size, Z = Z-statistics for a level of confidence (for 95% level of confidence, Z = 1.96), P = expected prevalence in population based on previous published studies [in proportion of 1, so, the estimated frequency of inappropriate high dosing in CKD patients was 74.0% or 0.74 [11], d = absolute error or precision (in proportion of 1, if 5%, d = 0.05). By putting these values in the abovementioned formula, the minimum number of patients required for this study was 295.

### Data collection and identification of medication dosing errors

To collect patients’ data, a standardized data collection form devised on the basis of extensive literature review and inputs from the clinical team at the study site and supervisory committee was used. The patients’ files, medication prescriptions and laboratory findings were used to gather the data. The data collected consisted of patient’s age, gender, weight, serum creatinine, stages of CKD, comorbidities, serum electrolytes and prescribed medications along with dose and frequency. Additionally, the number of prescribed medications lines and their generic names were recorded, and they were categorized according to their respective pharmacological classes [17, 24]. Since no national drug dosing guidelines for CKD exists in Pakistan, the appropriateness of medication doses was checked against various resources including renal drug handbook- 2018 [25], Kidney Disease Improving Global Outcome**s ***(*KDIGO) guidelines [1, 26, 27], British National Formulary-2022 [28], and manufacturer leaflets. The daily dosage of each medication was established by multiplying the unit dose (mg) and the frequency of administration. The reference documents listed above were used to determine the recommended daily dose (mg) based on the patient’s eGFR. Inappropriate high dosages were identified by comparing the prescribed drug’s daily dose to its recommended daily dose. Similar methodologies were used in other studies that investigated inappropriate high dosages among CKD patients [10, 11, 29, 30].

### Statistical analysis

The data were analysed using Statistical Package for Social Sciences (version 23). Multiple binary logistic regression analysis was used for evaluating the factors associated with receiving inappropriate high doses. After checking for collinearity, those independent variables which had a p-value < 0.20 in univariate analysis were entered in multivariate analysis. If two variables had a high collinearity (tolerance value < 0.10 and/or variance inflation factor > 10) one of them was excluded from the final model. A p-value < 0.05 was considered statistically significant.

## Results

### Socio-demographic and clinical characteristics of patients

A total of 365 ND-CKD patients were assessed for inclusion in the study. Of those patients, 325 met the established eligibility criteria, and 303 were included in the final analysis after giving consent. The mean age of study participants was 52.9 ± 14.0 years. Majority of them were males (56.4%), 41–60 years old (55.4%), had body weight of > 80 kg (55.4%) and suffered from CKD stage 5 (40.6%). Moreover, nearly all of the patients had at least one comorbidity (98.7%) with hypertension being the most common one (84.2%), followed by anaemia (78.2%) and diabetes mellitus (43.6%) (Table 1).

**Table 1:** Socio-demographic and clinical characteristics of patients

Variable	No. (%)	Mean ± SD
Gender		
Male	171 (56.4)	
Female	132 (43.6)	
**Age category** (years)		
< 40	63 (20.8)	
41–60	168 (55.4)	
> 60	72 (23.8)	
**Weight category** (kilogram)		
41–60	27 (8.9)	
61–80	108 (35.6)	
> 80	168 (55.4)	
**CKD stage**		
3a	12 (4.0)	
3b	63 (20.8)	
4	105 (34.7)	
5	123 (40.6)	
**Presence of one or more comorbidities**	299 (98.7)	
Number of comorbidities		
1	65 (21.5)	
2	99 (32.7)	
3	120 (39.6)	
4	15 (5.0)	
Hypertension	255 (84.2)	
Anaemia	237 (78.2)	
Diabetes mellitus	132 (43.6)	
Cardiovascular diseases	78 (25.7)	
Serum creatinine (mg/dl)		4.3 ± 2.9
Urea (mg/dl)		127.8 ± 81.1
Haemoglobin (g/dl)		10.1 ± 1.9
Serum sodium (mEq/L)		151.6 ± 133.1
Serum potassium (mEq/L)		4.6 ± 0.9
Serum chloride (mEq/L)		106.4 ± 5.6

CKD, chronic kidney disease; dl, decilitre; g, gram; mEq, milli-equivalent; mg, milligram; L, litre; SD, standard deviation

### Drugs prescription pattern

Table 2 displays the drug prescription pattern of study participants, wherein, a total of 2,265 medications were prescribed, with each patient receiving a median of eight different drugs (interquartile range 6–9 drugs).

**Table 2:** Drugs prescription pattern

Drugs prescribed	No. (%)
**Anti-acidotic agents**	267 (88.1)
**Antihypertensive drugs**	252 (83.2)
Renin-angiotensin system inhibitors	123 (40.6)
Calcium channel blockers	165 (54.5)
Beta-blockers	72 (23.8)
Diuretics	135 (43.6)
Alpha-blockers	25 (8.2)
**Vit-D preparations**	**215 (70.9)**
**Anti-anaemics**	**168 (55.4)**
**Anti-emetic**	**165 (54.5)**
**Antidiabetic drugs**	109 (36.0)
Biguanides	33 (10.9)
Sulfonylureas	42 (13.9)
Gliptins	54 (17.8)
**NSAIDs + tramadol**	**91 (30.0)**
**Lipid lowering agent (statins)**	**65 (21.4)**
**Anti-platelets**	**77 (25.4)**
**Proton pump inhibitors**	**95 (31.3)**
**Anti-psychotics**	**42 (13.9)**
**Multi-vitamins**	**46 (15.2)**
**Antibiotics**	**24 (7.9)**

ACEIs, angiotensin converting enzyme inhibitors; ARBs, angiotensin receptor blockers; NSAIDs, non-steroidal anti-inflammatory drugs

### Frequency and factors associated with drugs prescribed at inappropriate high doses

Based on the dosing guidelines, 34.5% (783/2265) of these drugs should have dosed according to the patients’ eGFR. However, among these 783 drugs, doses were not adjusted for 56.1% (440) of them in 53.4% (162) patients. The most common drugs not adjusted for dose based on patient’s eGFR were cefixime (100%) followed ciprofloxacin (83.3%), lisinopril (76.9%), bisoprolol (76.6%), glibenclamide (75.0%), losartan (70.3%), tramadol (68.0%), spironolactone (66.6%), aspirin (63.1%), metformin (60.6%) sitagliptin (60.6%), clarithromycin (57.1%), simvastatin (56.5%), domperidone (53.9%), captopril (52.0%) enalapril (51.8%), hydrochlorothizide (51.1%) and gliclazide (50.0%) (Table 3).

**Table 3:** Pattern and frequency of drugs with unadjusted doses

Drugs	Drugs which needed dose adjustment No.	Appropriate doses No. (%)	Frequency of inappropriate high doses			Inappropriate high doses No. (%)
			CKD stage 3 No.	CKD stage 4 No.	CKD stage 5 No.	
Antihypertensives						
**ACEIs**						**37 (56.9)**
Captopril	25	12 (48.0)		8	5	13 (52.0)
Enalapril	27	13 (48.1)		8	6	14 (51.8)
Lisinopril	13	3 (23.0)		4	6	10 (76.9)
**ARBs**						**32 (55.2)**
Losartan	27	8 (29.6)		15	4	19 (70.3)
Valsartan	31	18 (58.0)		9	4	13 (41.9)
**Beta blockers**						**41 (56.9)**
Atenolol	42	24 (57.1)		7	11	18 (42.8)
Bisoprolol	30	7 (23.3)		10	13	23 (76.6)
**Diuretics**						**77 (57.0)**
Hydrochlorothiazide	84	41 (48.8)		24	19	43 (51.1)
Spironolactone	51	17 (33.3)		18	16	34 (66.6)
**Antidiabetics**	108					**64 (59.2)**
Metformin	33	13 (39.3)	3	5	12	20 (60.6)
Sitagliptin	33	13 (39.3)	3	5	12	20 (60.6)
Gliclazide	14	7 (50.0)		3	4	7 (50.0)
Glimepiride	12	7 (58.3)		2	3	5 (41.6)
Glibenclamide	16	4 (25.0)		5	7	12 (75.0)
**Antihyperlipidimics**						**30 (46.1)**
Rosuvastatin	42	25 (59.5)		8	9	17 (40.4)
Simvastatin	23	10 (43.4)		5	8	13 (56.5)
**NSAIDs + analgesics**						**51 (56.0)**
Aspirin	19	7 (36.8)		8	4	12 (63.1)
Tramadol	47	15 (31.9)		15	17	32 (68.0)
Paracetamol	25	18 (72.0)		3	4	7 (28.0)
**Anti-emetics**						
Domperidone	165	76 (46.0)		39	50	**89 (53.9)**
**Antibiotics**						**19 (79.1)**
Cefixime	5	0		3	2	5 (100.0)
Ciprofloxacin	12	2 (16.6)		4	6	10 (83.3)
Clarithromycin	7	3 (42.8)		3	1	4 (57.1)

ACEIs, angiotensin converting enzyme inhibitors; ARBs, angiotensin receptor blockers; CKD, chronic kidney disease; NSAIDs, non-steroidal anti-inflammatory drugs

### Factors associated with inappropriate high dosing

The results of multivariate binary regression analysis revealed that patient’s age of 41–60 years (OR = 5.76, p-value < 0.001) and > 60 years (OR = 9.49, p-value < 0.001), suffering from hypertension (OR = 2.68, p-value = 0.012), diabetes mellitus (OR = 3.47, p-value < 0.001) and cardiovascular disease (OR = 2.82, p-value = 0.004) had statistically significant association with receiving inappropriate high doses. The fitness of this model was based on non-significant Hosmer Lemeshow (p-value = 0.386) overall percentage of 78.2% from classification table (Table 4).

**Table 4:** Factors associated with receiving inappropriate high doses

Variables	Inappropriate high dose Frequency (%)	Univariate analysis		Multivariate analysis	
		OR (95%CI)	p-value	OR (95%CI)	p-value
Gender					
Male	96 (56.1)	Referent			
Female	66 (50.1)	0.78 (0.49–1.23)	0.288	-	-
**Age** (years)					
≤ 40	9 (14.3)	Referent			
41–60	105 (61.4)	9.54 (4.42–20.61)	< 0.001	5.76 (2.46–13.50)	**< 0.001**
> 60	48 (69.6)	13.71 (5.73–32.81)	< 0.001	9.49 (2.87–21.56)	**< 0.001**
**Weight** (kilogram)					
41–60	6 (22.2)	Referent			
61–80	60 (55.6)	4.37 (1.64–11.70)	0.003	2.75 (0.84–8.93)	0.093
> 80	96 (57.1)	4.67 (1.79–12.16)	0.002	2.82 (0.87–9.12)	0.083
**CKD stage**					
3	6 (40.0)	Referent			
4	45 (42.9)	1.12 (0.37–3.39)	0.834	0.37 (0.07–1.89)	0.230
5	111 (60.7)	2.31 (0.79–6.77)	0.126	1.24 (0.26–5.96)	0.790
**Hypertension**					
No	18 (37.5)	Referent			
Yes	144 (56.5)	2.16 (1.15–4.08)	0.017	2.68 (1.24–5.80)	**0.012**
**Anemia**					
No	24 (36.4)	Referent			
Yes	138 (58.2)	2.44 (1.39–4.29)	0.002	1.90 (0.95–3.78)	0.067
**Diabetes mellitus**					
No	63 (36.8)	Referent			
Yes	99 (75.0)	5.14 (3.11–8.49)	< 0.001	3.47 (1.91–6.32)	**< 0.001**
**Cardiovascular disease**					
No	108 (48.0)	Referent			
Yes	54 (96.2)	2.44 (1.41–4.21)	0.001	2.82 (1.40–5.67)	**0.004**
**Other comorbidities**					
No	156(54.2)	Referent			
Yes	6 (40.0)	0.56 (0.20–1.63)	0.289	-	-

CI, confidence interval; CKD, chronic kidney disease; OR, odds ratio

## Discussion

This study evaluated the frequency, pattern and factors associated with inappropriate high doses in ND-CKD patients who received treatment at nephrology OPD of a tertiary care hospital in Pakistan. In order to make a distinction between the patients suffering from CKD and acute kidney injury (who need frequent dose adjustment), we included only established ND-CKD patients who were previously been treated for CKD at the study site or other hospitals or clinics. We observed that among the total prescribed drugs (n = 2265), 34.5% (n = 783) required dose adjustment, and only 45.9% (n = 440/783) drugs had their doses adjusted appropriately. The proportion of drugs with unadjusted doses (56.1%) in our study was within the range (13-84.3%) reported by studies conducted in Pakistan and elsewhere [7–10, 18]. The variable proportion of drugs with unadjusted doses in different studies could be due to differences in the study population (inpatients vs. outpatients), CKD stages, presence of comorbidities, the approaches and references used for evaluating dose appropriateness, prescribing pattern and availability of clinical pharmacists at the study sites [7–10, 18, 31]. The physicians’ lack of knowledge about the drugs requiring dose adjustment in CKD patients, underestimation of the potential adverse consequences [7–10, 18, 31, 32], and a lack of consensus on appropriate renal dosing guidelines for many drugs [7] could be some of the possible explanations for high proportion of drugs with unadjusted doses at the study site. Although the use of multiple reference documents for defining dose appropriateness was the strength of this study, but we did not evaluate the control of comorbidities, this could have been a potential cause of physicians’ hesitation to down-titrate the doses of drugs during the clinical encounters, contributing to the high prevalence of inappropriate dosing at the study site. However, it is also important to acknowledge that the use of a daily dose cut-off value criterion to define inappropriate high doses in our study may have resulted in an underestimation of the overdose rate.

Although, antibiotics (cefixime, ciprofloxacin and clarithromycin) comprised only 7.9% (24/2265) of the total prescribed drugs in our study, we found that 79.1% (19/24) of them were not adjusted for dose. The inappropriate dosing of these drugs in patients with CKD may lead to acute renal failure with tubulointerstitial nephritis [33, 34], neurotoxicity (hallucination and psychosis) [35] and cardiotoxicty (QT interval prolongation and sudden cardiac death) [35]. In our study, metformin and sitagliptin were the two most frequently prescribed antidiabetic agents, followed by glibenclamide, gliclazide and glimepiride. However, it was noted that doses of 59.2% of the prescribed antidiabetics [metformin (60.6%), sitagliptin (60.6%), gliclazide (50.0%), glimepiride (41.6%) and glibenclamide (75.0%)] were not adjusted appropriately. This is concerning as the use of metformin in CKD patients with concurrent hypoxemic conditions may lead to lactic acidosis [3], and it is recommended that the dose of metformin should be reduced by 50% in CKD stage-3, and should be avoided altogether in CKD stage 4 and 5 patients [36]. Additionally, the plasma concentration of sitagliptin can increase by two to four folds in patients with moderate to severe CKD, increasing the risk congestive heart failure [37, 38]. Therefore, it is recommended to start sitagliptin at a lower dose in patients with an eGFR < 45 ml/min/1.73 m^2^ [39]. Glibenclamide, gliclazide and glimepiride are long acting potent hypoglycemic drugs. Their use without dose adjustment in patients with renal impairment could result in episodes of severe hypoglycaemia [1]. The renin-angiotensin system inhibitors (ACEIs and ARBs) due to their antiproteinuric, nephroprotective and cardioprotective effects and halting the progression of renal impairment are preferred agents in CKD patients. However, it is concerning that in our study doses of captopril (52.0%), enalapril (51.8%), lisinopril (76.9%), losartan (70.3%) and valsartan (41.9%) were not adjusted in CKD patients. The use of these agents without dose adjustment in CKD stage 4 and 5 patients, particularly in the presence of congestive heart failure and non-steroidal anti-inflammatory drugs (NSAIDs), could cause acute renal failure and hyperkalemia [27, 40–43]. Additionally, our study found that doses of bisoprolol (76.6%) and atenolol (42.8%) were not adjusted. As these agents are hydrophilic and mainly excreted through the kidney, their use without dose adjustment could result in drug induced cellular injury, inflammation in the renal interstitium and acute renal failure in CKD patients [3, 44, 45]. The analysis further revealed that doses were also not adjusted for hydrochlorothiazide (56.1%) and spironolactone (66.6%). It is worth noting that hydrochlorothiazide has been reported to be ineffective in patients with eGFR < 30 ml/min/1.73m^2^, and its use in CKD stage 4 and 5 patients could lead to severe episodes of hyponatremia, hypokalaemia, volume depletion and acute kidney injury [46, 47]. On the other hand, the inappropriate high doses of spironolactone in patients with eGFR < 30 ml/min/1.73m^2^ could result in severe hyperkalaemia and increase the absolute risk of worsening kidney function [46, 47]. As the excretion of domperidone decreases with decline in kidney function, the repeated doses can increase its plasma half-life in CKD stage 4 and 5 patients. Its prescription with unadjusted dose (53.9% in the current study) could result in QT-interval prolongation and increased risk of sudden cardiac death in CKD patients suffering from cardiovascular diseases [48]. The study further showed that inappropriate high doses of aspirin and paracetamol were prescribed to 43.1% patients who were receiving them. As these agents inhibit prostaglandin-E_2_ which can result in vasoconstriction and a decrease in renal blood flow, they can potentially worsen the renal function. Therefore it is advised to avoid these agents until absolutely necessary. If their use is unavoidable, treatment should be started with the minimum effective doses, and they should only be used for the shortest possible period [49]. The prescription tramadol without dose adjustment could result in its extended plasma half-life and substantial adverse effects on central nervous system [50].

The multivariate analysis in our study revealed that patients over the age of 40 years and those with hypertension, diabetes mellitus and cardiovascular diseases were at significantly high risk of receiving inappropriate high doses of drugs. Our findings are consistent with previous studies that have reported older age as a risk factor for receiving inappropriate high doses of renally cleared drugs [10, 12]. While age related deterioration in renal function should warrant dose reduction or avoidance of nephrotoxic drugs even in the absence of CKD, unfortunately, these factors are often not taken in account when prescribing medications [7, 12]. Likewise, our study findings are consistent with previous research which suggests that comorbidities like hypertension, diabetes mellitus and cardiovascular diseases can increase the risk of CKD patients receiving inappropriate high doses of medications [7, 10–12]. Given that these comorbidities are often present in CKD patients and can contribute to renal impairment, therefore, these patients treated with multiple drugs are at greater risk of receiving inappropriate high doses [12].

Although our study had several strengths, including a sufficient sample size, the multiple reference documents to assess the appropriate drug dosage, and the use of eGFR for estimating renal function, there are some limitations that must be acknowledged. First our study was conducted at a single centre, which may limit the generalizability of our results to other settings. Our participants were selected using convenience sampling technique, which may not have been the representative of the entire CKD population. We also could not evaluate the potential consequences of inappropriate high dosing on patients’ clinical outcomes. The use of single serum creatinine value for calculating eGFR could have under or overestimated renal function. Furthermore, the physicians at our study site may have considered other parameters such as blood pressure, blood glucose, cholesterol, or serum electrolytes levels when making dose adjustments, which we were unable to account for in our analysis. It is also possible that different dosing guidelines were used for dose adjustment than those utilized in our study. Therefore, to confirm the findings of our study and overcome these limitations, a large multicentre study that considers a wider range of factors affecting medication dosing in CKD patients is recommended.

## Conclusion

The study findings highlight a significant quality gap in medication dosing for patients with ND-CKD at the study site, as inappropriate doses were prevalent, and most drugs requiring dose adjustment were not appropriately adjusted. To address this issue, the continued medical education of clinical pharmacokinetics for doctors, the calculation of GFR by the support staff prior to prescription, drug dosing services provided by clinical pharmacists, and the display of charts of drugs requiring dose adjustment in CKD patients may help to reduce the frequency of inappropriate dosing. Special attention should be paid to the drugs and patients with identified risk factors for inappropriate high dosing.

## Data Availability

All data gathered or analyzed during this study are included in the article. The raw data on which conclusions of this manuscript is based is available upon request. Please contact Nafees Ahmad at nafeesuob@gmail.com.

## Abbreviations

ACEIs: Angiotensin converting enzymes inhibitors
ARBs: Angiotensin receptor blockers
CI: Confidence interval
CKD: Chronic kidney diseases
eGFR: Estimated glomerular filtration rate
ND: Non-dialysis
NSAIDs: Non-steroidal anti-inflammatory drugs
OPD: Outpatients department
OR: Odds ratio
SPH: Sandeman Provincial Hospital

## References

[1] Rossing P, Caramori ML, Chan JC, Heerspink HJ, Hurst C, Khunti K (2022). KDIGO 2022 clinical practice guideline for diabetes management in chronic kidney disease. Kidney Int.

[2] Lameire NH, Levin A, Kellum JA, Cheung M, Jadoul M, Winkelmayer WC et al. Harmonizing acute and chronic kidney disease definition and classification: report of a Kidney Disease: Improving Global Outcomes (KDIGO) Consensus Conference. *Kidney Int*. 2021;100(3):516–526. 10.1016/j.kint.2021.06.028 34252450

[3] Munar MY, Munar MY, Signh H (2007). Drug dosing adjustments in patients with chronic kidney disease. Am Fam Physician.

[4] Hassan Y, Al-Ramahi RJ, Aziz NA, Ghazali R (2009). Drug use and dosing in chronic kidney disease. Ann Acad Med Singap.

[5] Nolin TD. A synopsis of clinical pharmacokinetic alterations in advanced CKD.Semin Dial.2015;325–329. 10.1111/sdi.12374 PMC4496305 25855244

[6] Mason NA (2011). Polypharmacy and medication-related complications in the chronic kidney disease patient. Curr Opin Nephrol Hypertens.

[7] Chang F, O’Hare AM, Miao Y, Steinman MA (2015). Use of renally inappropriate medications in older veterans: a national study. J Am Geriatr Soc.

[8] Bilge U, Sahin G, Unluoglu I, Ipek M, Durdu M, Keskin A (2013). Inappropriate use of nonsteroidal anti-inflammatory drugs and other drugs in chronic kidney disease patients without renal replacement therapy. Ren Fail.

[9] Roux-Marson C, Baranski JB, Fafin C, Exterman G, Vigneau C, Couchoud C (2020). Medication burden and inappropriate prescription risk among elderly with advanced chronic kidney disease. BMC Geriatr.

[10] Saleem A, Masood I (2016). Pattern and predictors of medication dosing errors in chronic kidney disease patients in Pakistan: a single center retrospective analysis. PLoS ONE.

[11] Getachew H, Tadesse Y, Shibeshi W (2015). Drug dosage adjustment in hospitalized patients with renal impairment at Tikur Anbessa specialized hospital, Addis Ababa, Ethiopia. BMC Nephrol.

[12] Khanal A, Peterson GM, Castelino RL, Jose MD (2015). Potentially inappropriate prescribing of renally cleared drugs in elderly patients in community and aged care settings. Drugs Aging.

[13] Watanabe JH, McInnis T, Hirsch JD (2018). Cost of prescription drug–related morbidity and mortality. Ann Pharmacother.

[14] Tesfaye WH, Castelino RL, Wimmer BC, Zaidi STR (2017). Inappropriate prescribing in chronic kidney disease: a systematic review of prevalence, associated clinical outcomes and impact of interventions. Int J Clin Pract.

[15] Chaudhry A. Alarming increase in end-stage kidney disease.Dawn [newspaper on the Internet]2017.

[16] Hasan M, Sutradhar I, Gupta RD, Sarker M (2018). Prevalence of chronic kidney disease in South Asia: a systematic review. BMC Nephrol.

[17] Gulalai, Ahmad N, Wahid A, Khan A, Atif M, Khan A (2020). Evaluation of management and factors associated with hypertension control in hemodialysis patients at a tertiary-care hospital in Pakistan. Drugs Ther Perspect.

[18] Hassan Z, Ali I, Ullah AR, Ahmed R, Zar A, Ullah I et al. Assessment of medication Dosage Adjustment in Hospitalized Patients with Chronic Kidney Disease.Cureus.2021;13(2). 10.7759/cureus.13449 PMC7982869 33767933

[19] Akbar Z, Rehman S, Khan A, Khan A, Atif M, Ahmad N (2021). Potential drug–drug interactions in patients with cardiovascular diseases: findings from a prospective observational study. J Pharm Policy Pract.

[20] Abdullah A, Ahmad N, Atif M, Khan S, Wahid A, Ahmad I et al. Treatment Outcomes of Childhood Tuberculosis in Three Districts of Balochistan, Pakistan: Findings from a Retrospective Cohort Study.J Trop Pediatr. 2020. 10.1093/tropej/fmaa042 32647882

[21] Florkowski CM, Chew-Harris JS (2011). Methods of estimating GFR–different equations including CKD-EPI. Clin Biochem Rev.

[22] Ferraro PM, Lombardi G, Gambaro G. Impact of the new, race-free CKD-EPI equation on prevalence and clinical outcomes of CKD in northeastern Italy: the INCIPE study.J Nephrol.2022:1–3. 10.1007/s40620-022-01284-2 35175579

[23] Murton M, Goff-Leggett D, Bobrowska A, Garcia Sanchez JJ, James G, Wittbrodt E (2021). Burden of chronic kidney disease by KDIGO categories of glomerular filtration rate and albuminuria: a systematic review. Adv Ther.

[24] Ahmad N, Hassan Y, Tangiisuran B, Meng OL, Aziz NA, Khan AH (2012). Guidelines adherence and hypertension control in an outpatient cardiology clinic in Malaysia. Trop J Pharm Res.

[25] The Renal Drug Handbook, vol. 5th edition 5th edn: CRC Press is an imprint of the Taylor & Francis Group, an informa business. ; 2018. Available at; https://www.medicinainterna.net.pe/sites/default/files/The_Renal_Drug_Handbook_The_Ultimate.pdf. Accesed 10 November 2022.

[26] Kidny Disease Improving Global Outcomes. Blood pressure guideline. 2021; Available at: https://kdigo.org/wp-content/uploads/2016/10/KDIGO-2021-BP-GL.pdf. Accessed 10 November 2022.

[27] Kidny Disease Improving Global Outcomes. Anemia guideline. 2012; Available at: https://kdigo.org/wp-content/uploads/2016/10/KDIGO-2012-Anemia-Guideline-English.pdf. Accessed 20 November 2022.

[28] British National Formulary 83 March –. September 2022; Available at: https://nhathuocngocanh.com/wp-content/uploads/2022/05/BNF-83-British-National-Formulary-in-2022.pdf. Accessed 8 December 2022.

[29] Sweileh WM, Janem SA, Sawalha AF, Abu-Taha AS, Zyoud SeH, Sabri IA (2007). Medication dosing errors in hospitalized patients with renal impairment: a study in Palestine. Pharmacoepidemiol Drug Saf.

[30] Sheen SS, Choi JE, Park RW, Kim EY, Lee YH, Kang UG (2008). Overdose rate of drugs requiring renal dose adjustment: data analysis of 4 years prescriptions at a tertiary teaching hospital. J Gen Intern Med.

[31] Alahdal AM, Elberry AA (2012). Evaluation of applying drug dose adjustment by physicians in patients with renal impairment. Saudi Pharm J.

[32] Dhib M, Moulin B, Leroy A, Hameau B, Godin M, Johannides R (1991). Relationship between renal function and disposition of oral cefixime. Eur J Clin Pharmacol.

[33] Ma TK-W, Chow K-M, Choy ASM, Kwan BC-H, Szeto C-C, Li PK-T (2014). Clinical manifestation of macrolide antibiotic toxicity in CKD and dialysis patients. Clin Kidney J.

[34] Connor JP, Curry JM, Selby TL, Perlmutter AD (1994). Acute renal failure secondary to ciprofloxacin use. J Urol.

[35] Saher T, Al-Worafi YM, Iqbal MN, Wahid A, Iqbal Q, Khan A (2022). Doctors’ adherence to guidelines recommendations and glycaemic control in diabetic patients in Quetta, Pakistan: findings from an observational study. Front Med.

[36] Wu S, Hopper I, Skiba M, Krum H (2014). Dipeptidyl peptidase-4 inhibitors and cardiovascular outcomes: meta‐analysis of randomized clinical trials with 55,141 participants. Cardiovasc Ther.

[37] Wang K-L, Liu C-J, Chao T-F, Huang C-M, Wu C-H, Chen S-J (2014). Sitagliptin and the risk of hospitalization for heart failure: a population-based study. Int J Cardiol.

[38] Muanda FT, Weir MA, Bathini L, Clemens KK, Perkovic V, Sood MM (2020). Higher-dose sitagliptin and the risk of congestive heart failure in older adults with CKD. Clin J Am Soc Nephrol.

[39] McCoy IE, Han J, Montez-Rath ME, Chertow GM. Barriers to ACEI/ARB use in proteinuric chronic kidney disease: An observational study. Mayo Clin Proc: 2021:Elsevier. 2021;pp. 2114–2122. 10.1016/j.mayocp.2020.12.038 PMC8973200 33952396

[40] Sica DA (2003). Renal handling of angiotensin receptor blockers: clinical relevance. Curr Hypertens Rep.

[41] Whelton P, Carey R, Aronow W. ACC/AHA/AAPA/ABC/ACPM/AGS/APHA/ASH/ASPC/NMA/PCNA guideline for the prevention, Detection, evaluation, and management of high blood pressure in adults: a Report of the American College of Cardiology/American heart Association. Task force on clinical practice guidelines. *J Am Coll Cardiol*. 2017.-Nov 13. Почки 2018, 7(1):68–74. 10.1016/j.jacc.2017.11.006 29146535

[42] UK NCGC. : Investigating chronic kidney disease. Chronic Kidney Disease (Partial Update): Early Identification and Management of Chronic Kidney Disease in Adults in Primary and Secondary Care.edn.: National Institute for Health and Care Excellence (UK); 2014. 25340245

[43] Tomiyama H, Yamashina A. Beta-blockers in the management of hypertension and/or chronic kidney disease.Int J Hypertens. 2014;2014. 10.1155/2014/919256 PMC3941231 24672712

[44] Pannu N, Nadim MK (2008). An overview of drug-induced acute kidney injury. Crit Care Med.

[45] Initiative KDOQ (2004). K/DOQI clinical practice guidelines on hypertension and antihypertensive agents in chronic kidney disease. Am J Kidney Dis.

[46] Beldhuis IE, Myhre PL, Claggett B, Damman K, Fang JC, Lewis EF (2019). Efficacy and safety of spironolactone in patients with HFpEF and chronic kidney disease. JACC: Heart Fail.

[47] Domperidone (1988). An alternative to metoclopramide. Drug Ther Bull.

[48] Rossi M, Giorgi G (2010). Domperidone and long QT syndrome. Curr Drug Saf.

[49] Neerkin J, Brennan M, Jamal H. Use of opioids in patients with impaired renal function. In.: Nottingham, Engl: Palliativedrugs. com;2012.

[50] Han Y, Balkrishnan R, Hirth RA, Hutton DW, He K, Steffick DE (2020). Assessment of prescription analgesic use in older adults with and without chronic kidney disease and outcomes. JAMA Netw Open.

